# Retraction: Suppression of uPA and uPAR Attenuates Angiogenin Mediated Angiogenesis in Endothelial and Glioblastoma Cell Lines

**DOI:** 10.1371/journal.pone.0330088

**Published:** 2025-08-14

**Authors:** The PLOS One Editors

After the publication of this article [1], concerns were raised with results presented in Fig 4. Specifically,

- Lanes 3-5 of the Fig 4G HMEC GAPDH panel appear to overlap with lanes 1-3 of the Fig 4I HMEC GAPDH panel.
- The Fig 4H HMEC GAPDH panel of this article [1] appears similar to the Fig 2A 5310 GAPDH panel of [2].
- Following the publication of this article [1], the U87 MG cell line used in this study was identified as a misidentified cell line [3].
- The animal ethics approval reported in this study may not have been obtained prospectively.

In addition to the issues listed above, concerns were raised about the validity of primers used in the RT-PRC experiment.

The authors did not respond to the journal’s communications, and the concerns remain unresolved.

The unresolved concerns call into question the reliability and validity of the published results, and the article’s adherence to the journal’s editorial policies in place at the time the article was submitted. In light of these unresolved concerns, the *PLOS One* Editors retract this article.

All authors either did not respond directly or could not be reached.
